# Fabrication and characterization of zeolitic imidazolate framework-embedded cellulose acetate membranes for osmotically driven membrane process

**DOI:** 10.1038/s41598-019-42235-5

**Published:** 2019-04-08

**Authors:** Teayeop Kim, Moon-ki Choi, Hyun S. Ahn, Junsuk Rho, Hyung Mo Jeong, Kyunghoon Kim

**Affiliations:** 1 School of Mechanical Engineering, Sungkyunkwan University, Suwon, 16419 Republic of Korea; 2 Department of Chemistry, Yonsei University, Seoul, 03722 Republic of Korea; 3 Department of Chemical Engineering, Pohang University of Science and Technology, Pohang, 790-784 Republic of Korea; 4 Department of Materials Science & Engineering, Kangwon National University, Chuncheon, 24341 Republic of Korea

## Abstract

Zeolitic imidazolate framework-302 (ZIF-302)-embedded cellulose acetate (CA) membranes for osmotic driven membrane process (ODMPs) were fabricated using the phase inversion method. We investigated the effects of different fractions of ZIF-302 in the CA membrane to understand their influence on ODMPs performance. Osmotic water transport was evaluated using different draw solution concentrations to investigate the effects of ZIF-302 contents on the performance parameters. CA/ZIF-302 membranes showed fouling resistance to sodium alginate by a decreased water flux decline and increased recovery ratio in the pressure retarded osmosis (PRO) mode. Results show that the hydrothermally stable ZIF-302-embedded CA/ZIF-302 composite membrane is expected to be durable in water and alginate-fouling conditions.

## Introduction

Water purification is an important issue because of growing populations and energy demands that cause increasing water scarcity and environmental pollution^1–4^. To overcome global water scarcity, many researchers focus on water purification technologies such as distillation, sedimentation, and filtration^5^. Among these technologies, filtration using membranes is of much interest because of its low energy consumption^6^. Among membrane separation technology, osmotically driven membrane process (ODMP), is an effective strategy which harvests clean water across a semi permeable membrane and rejects a wide range of pollutants, including small ions, molecules and microorganisms. A type ODMP for desalination, forward osmosis (FO) process has lower fouling propensity attributed to low or lack of hydraulic pressure^7–10^. Among these advantage, ODMP wasn’t limited to desalination, but also proposed to pretreatment for reverse osmosis (RO)^9^ and thermal desalination^11,12^ which doesn’t require salt consumption. On the other hand, as an energy harvesting application of ODMP, pressure retarded (PRO) osmosis (PRO) process produces renewable energy from osmotic gradient between seawater and fresh water^13^. In order to improve the productivity of ODMP, various studies have focused on high water permeability, low solute flux, anti-fouling properties and water stability. In this regard, different kinds of membranes such as thin film composite, thin film nanocomposite, and cellulose acetate (CA) have been developed^14^. One of the conventional polymeric membranes, the CA membrane, has several advantages such as low cost, high fouling resistance and naturally existing monomers^15^. Many studies have optimized the process for fabricating CA membranes^15–18^. To enhance the membrane’s performance and ion-rejection ratio, many researchers have focused on incorporating nanomaterials into the membrane. The emergence of various nanomaterials serving as the nanosized particles (graphene oxide, carbon nanotubes, porous frameworks, titanate nanotubes, and SiO_2_) enable the membrane’s enhanced water permeability and can provide new solutions for fabricating advanced membranes for water purification^19–23^.

One of these nanomaterials, metal organic frameworks (MOFs), has been raised as a promising candidate for various applications in gas separation and storage. This is due to its highly selective gas adsorption through its own nanopores^24–28^. For the MOFs, various types of inorganic and organic linkers can construct 3D structures of particles which allows for a myriad of pore sizes and functionality type^29^. Compared to organic and inorganic porous nanomaterials, MOFs has advantage at uniformity pore size and functionality type within order which enables molecular transport with own selectivity^30^. Their unique selectivity has also been applied to desalination and separation technologies^31–34^. Various MOF functionalities can improve membrane permeation in different ways. However, the one of main problems with MOFs are their stability in water^35^. Among the MOF family, the zeolitic imidazolate framework (ZIF) has significant hydrothermal stability in water^35–38^. Because of its hydrothermal stability, ZIFs were applied to various types of desalination membrane. Furthermore, hydrophobic ZIF membranes also show substantial permeability in molecular dynamics simulations because its aperture size and hydrophobic functional group helps water pass through the membrane^30^.

**Figure 1:**
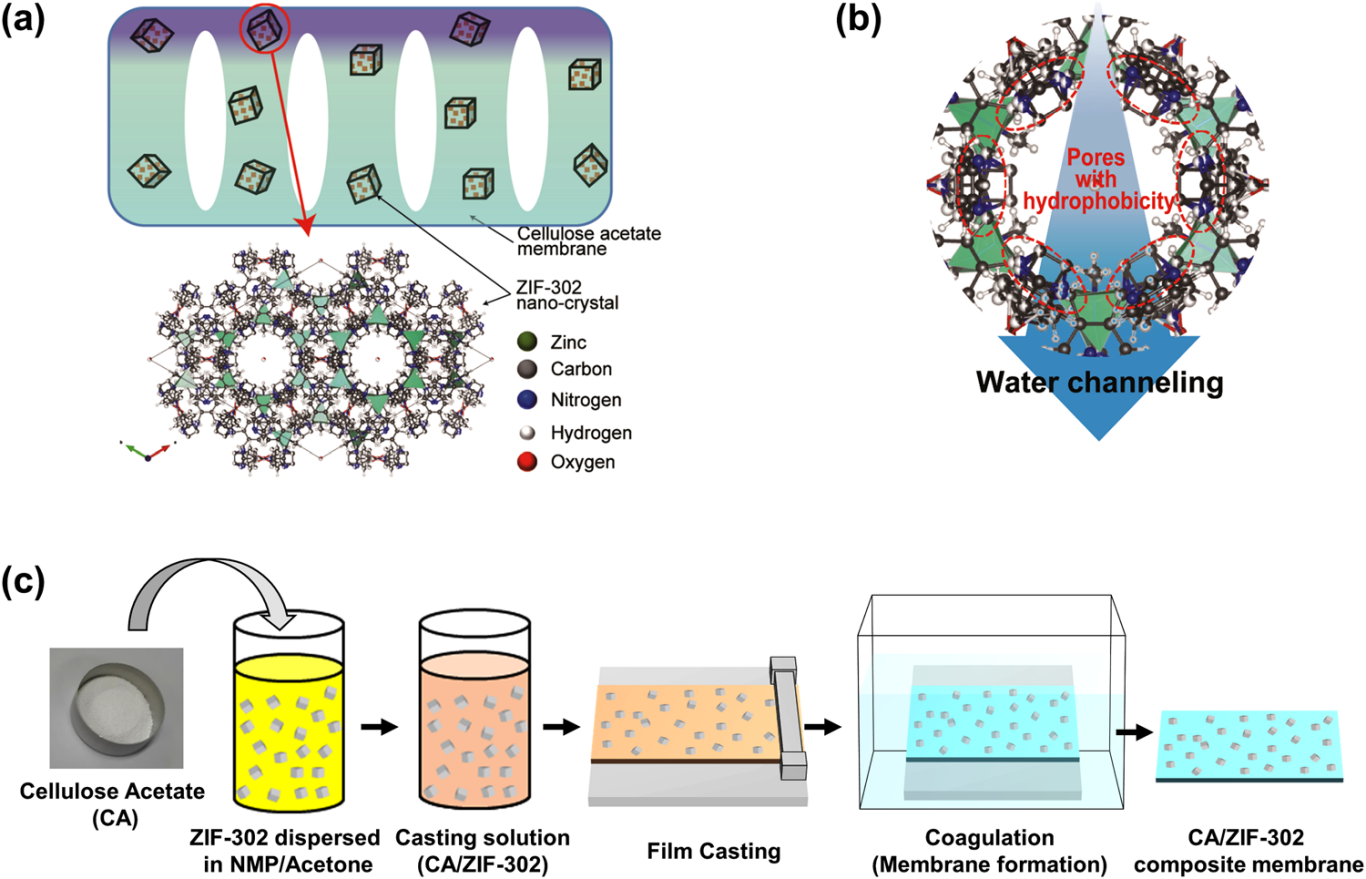
(**a**) Nano-enhanced ZIF-302/CA membrane. (**b**) Effects of hydrophobic pores in ZIF-302 (**c**) a schematic description of ZIF-302/CA membrane fabrication.

In this study, we focus on incorporating ZIF-302 crystals within the CA membrane to improve the membrane’s osmotic water flux (Fig. 1a). ZIF-302 can filter gases and other substances under both dry and humid conditions^36^. Also hydrophobicity of ZIF-302 lead to faster water transport by low interaction to water^39^ and rapid decay of hydrogen bonding^30^, while reject charged ion by energy barrier^40^ through hydrophobic channels (Fig. 1b). Chabazite structure of ZIF-302 with channel size of ~0.79 nm^41^ is correspond to pore diameter of osmosis membrane^42^ (0.6–0.8 nm) that enable selective transport of water molecules (0.28 nm) in brackish water. This approach applying ZIF-302 has difference in channel size (~0.79 nm) compared to ZIF-8 (0.34 nm) applied in previous studies.

The pore hydrophobicity that enables fast water transport by its weak interaction with water. The substantial hydrothermal resistance of ZIF-302 nano-enhanced materials play a key role in improving the CA membranes^36^. We investigated the effects of different ZIF-302 fractions and sizes in CA membranes. The CA/ZIF-302 composite membranes were simply fabricated by dispersion in solution followed by phase inversion process (Fig. 1c). Fabricated CA/ZIF-302 composite membranes were evaluated using laboratory-scale forward osmosis tests for water flux and reverse ion flux. Additional tests evaluated the effects of ZIF-302 contents on the viability of the membrane under harsh alginate fouling conditions. Our study aims to determine a ZIF-302 application to improve osmotic water transport of commercial CA membrane.

## Results and Discussion

### Characterization of ZIF-302 crystals and composite membranes

**Figure 2:**
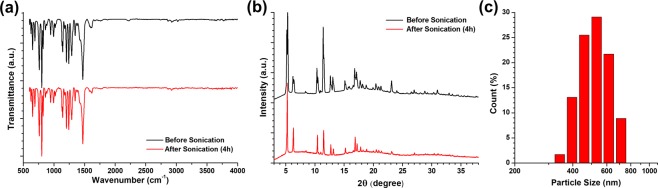
(**a**) FTIR spectra and (**b**) the XRD pattern of ZIF-302 crystals and (**c**) particle size distribution by intensity of ZIF-302 after ultrasonic process.

The water stability of MOFs has been an important issue in separation technologies with humid or wet conditions. Most types of MOFs are unstable in water because of the metal oxide clusters’ interactions with water^35,43^. While, ZIF-302 which is a type of MOFs showed hydrothermal stability which maintains its own crystallinity for 7 days in water at 100 °C^36^. In this study, the FTIR spectra and XRD patterns of ZIF-302 were analyzed before and after the ultrasonic process. After the ultrasonic process, using FTIR spectra and XRD patterns, the ZIF-302 nanocrystals showed that they maintained chemical structure and crystallinity, both showing similar peaks (Fig. 2). As-synthesized ZIF-302 crystals, which have sizes up to a few hundred micrometers, were split into nanocrystals after 4 h of ultrasonic processing. Adding ZIF-302 nanocrystals, CA/ZIF-302 composite membranes were fabricated with each different material composition showed in Table 1.

**Table 1:** Material composition of CA and CA/ZIF-302 membranes.

Membrane	Material composition	
	ZIF-302 (wt%)	CA (wt%)
CA	0	100
CZ5	5	95
CZ10	10	90
CZ15	15	85

**Figure 3:**
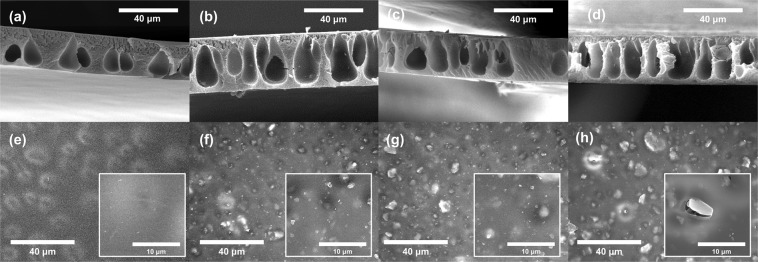
SEM image of (**a**–**d**) cross section and (**e**–**h**) top surface of membranes (**a**,**e**: CA, **b**,**f**: CZ5, **c**,**g**: CZ10, **d**,**h**: CZ15).

All membranes showed a dense top layer, which excludes ions, and were formed by rapid evaporation of acetone and solvent outflow during the coagulation process^15^. This dense layer, known as the selective active layer of the CA membrane, conducts osmotic water transport through the nanopores. The pure water permeability and ion permeability are dominantly determined by the active layer on the top surface, while the structural parameter influences the internal concentration polarization (ICP) in the ODMP, and is determined by the microstructure of the porous support layer. The structural parameter indicates effective diffusion length through membrane support layer^44^. The lower value of structural parameter is needed to achieve higher water flux as reducing ICP. In phase inversion membrane, microstructures of void have been divided to two cases, a sponge-like structure, which intensifies the ICP by its highly tortuous structure, and finger-like macro-voids (FLVs), which reduces the ICP. The sponge-like structure occupied most of the support layer, and only a few FLVs were in the bare CA membrane (Fig. 3a). However, more numerous and longer vertical FLVs were formed in the composite membrane support layers (Fig. 3b–d). The large ZIF particles found on the surface of the FLVs (Fig. 3b–d) seem to be an agglomeration of non-incorporated ZIF-302 nanocrystals because of their poor water dispersion.

The bare CA membrane shows a uniform and dense top surface (Fig. 3e), while ZIF-302 particles are found on the top surface of composite membranes (Fig. 3f–h). Figure 3f shows the top surface of the CZ5 membrane with many well-dispersed ZIF-302 nanocrystals and some microcrystals significantly larger than what DLS analysis showed in Fig. 2c. These incorporated particles in active layer improve water permeability as providing alternative flow paths to water molecules^45^. Since a larger agglomeration is detected in the composite membranes with high-concentrations of ZIF-302 contents, the number of incorporated ZIF particles is not proportional to the contents of ZIF-302. Even the defects around the large ZIF-302 particles could cause severe problems for membrane performance as far as direct ion permeation is concerned. The excessive ZIF-302 contents spoiled the membrane integrity by hindering solution transport during the coagulation process.

### Performance of ZIF-302/CA composite membranes

**Figure 4:**
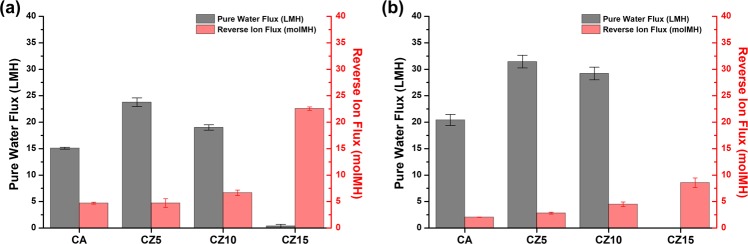
Pure water flux and reverse ion flux of the membrane in PRO mode with (**a**) 1 M NaCl and (**b**) 1 M MgCl_2_ DS (FS: DI water).

For ODMP, the membranes are required to achieve higher water flux and a lower reverse ion flux. First, for each membrane, both of these fluxes were measured in PRO mode with 1 M NaCl DS to investigate the influence of the ZIF-302 contents on the membrane (Fig. 4). The CZ5 membrane, with 5 wt% of ZIF-302 contents, showed 57% and 54% enhancements in the water flux when compared to a CA membrane in 1 M NaCl and 1 M MgCl_2_ DS, respectively. Water flux in the CZ5 membrane is improved by the nanocrystal incorporated active layer and microstructure of the support layer. As shown in Fig. 3e, as alternative flow path, individual crystals incorporated on active layer could improve osmotic water transport^21,33^. In support layer of CZ5 membrane, compared to CA membrane, a larger number of FLVs formed and they extended to vertical direction. These FLVs improve the water flow by reducing the ICP, and more effective, especially for long vertical shape^46,47^. On the contrary, CZ10 and CZ15 membranes with higher ZIF-302 contents did not demonstrate further water flux enhancements; they only exhibited higher reverse ion fluxes. Furthermore, there was no osmotic water flux in the CZ15 membrane. The excessive loading of the ZIF-302 nanocrystals might interfere with the formation of a uniform top layer and block mass transport. It is reported in previous works that excessive contents of nanomaterials reduce the ion rejection ratio of the active layer^21^. The active layer with low ion exclusion, which intensifies ICP, caused low osmotic water flux. As shown in Fig. 3g,h, more than 5 wt% of ZIF-302, result in larger numbers of aggregated crystals, and CZ15 membrane has large defects in top surface. The undesired defect formation via aggregation also occurred in previous studies because of excessive nanomaterial contents such as Ag nanoparticles^48^ and CNTs^49^. These aggregated crystals and large defects in active layer cause the decrease of ion exclusion and osmotic water flux. Although CZ10 and CZ15 contain large number of FLVs in support layer, which reduce ICP propensity, the active layer with low ion rejection causes low osmotic water flux. CZ15 membrane showed no functionality of active layer and little osmotic water flux due to large agglomeration and defects, through which ions permeate freely.

**Figure 5:**
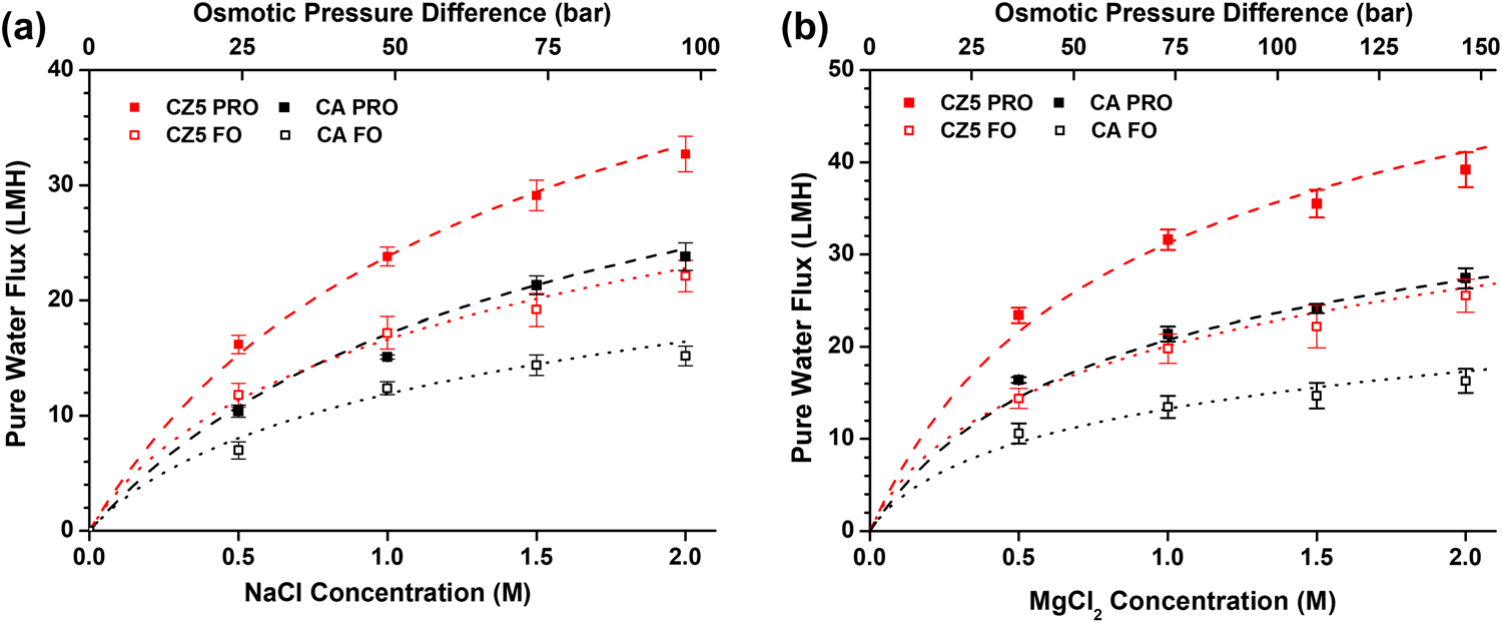
Pure water flux of membranes with (**a**) NaCl and (**b**) MgCl_2_ draw solutions at different concentrations.

There are two main modes for ODMP, the PRO mode and the FO mode. Although the PRO mode has a higher water flux than the FO mode, the FO mode has the advantage of a lower energy lower fouling propensity. Therefore, we investigated the osmotic water flux of the CA and CZ5 membranes using NaCl and MgCl_2_ DSs with different concentrations (Fig. 5). As a draw solute, MgCl_2_ showed a higher rejection ratio than NaCl (Fig. 4). In the ODMP, the higher ion rejection rate drove a higher water flux by reducing the ICP. As shown in Fig. 5, 1 M MgCl_2_ has the same osmotic pressure as 1.5 M NaCl, but the water flux was higher for 1 M MgCl_2_ DS than for 1.5 M NaCl in both the CA and CZ5 membranes. The pure water flux enhancement for the CZ5 membrane was higher at 1 M MgCl_2_ DS (48% in PRO mode, 47% in FO mode) than for 1.5 M NaCl DS (37% in PRO mode, 33% in FO mode).

The Table 2 shows calculated parametric transport properties of the CA and CZ5 membranes from experimental results. The CA membranes showed pure water permeability of 1.18 lm^−2^ h^−1^/bar and reverse salt permeability of 10.7 and 6.4 lm^−2^ h^−1^. Compared to bare CA membrane, CZ5 membrane which containing 5 wt% ZIF-302 contents showed 47% improved pure water permeability. The improvement of water permeability could be explained by the additional alternative path of water transport from incorporated ZIF-302 nanocrystals. The CZ5 membrane showed a significantly reduced structural parameter of 35% (336 ± 28 μm) compared to the CA membrane (520 ± 28 μm). The longer and larger number of FLVs in the CZ5 membrane may be attributed to the reduced structural parameter, which previous work has shown to be true^47,50^. The CZ5 membrane exhibited similar tendencies to the nanoparticle contents in casting solutions, reducing structural parameters by modified geometry as show in previous works^21,47^.

**Table 2:** Transport properties of membranes in terms of A, B, and S.

Membranes	Water permeability A (lm^−2^ h^−1^/bar)	NaCl permeability B_NaCl_ (lm^−2^ h^−1^)	MgCl_2_ permeability B_MgCl2_ (lm^−2^ h^−1^)	Structural Parameter S (μm)
CA	1.18 ± 0.05	10.7 ± 1.55	6.4 ± 0.46	520 ± 20
CZ5	1.72 ± 0.12	16.8 ± 2.93	10.2 ± 1.19	336 ± 28

### Alginate fouling test

**Figure 6:**
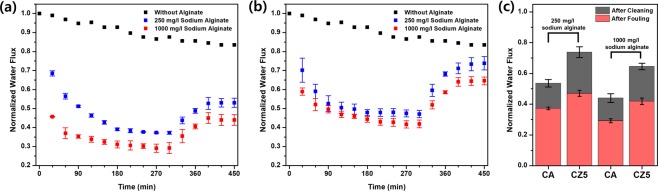
Normalized water flux of (**a**) the CA membrane and (**b**) the CZ5 membrane under alginate fouling conditions (PRO mode, DS: 1 M NaCl, FS: sodium alginate solution).

Sodium alginate is a hydrophilic natural organic substance used to investigate membrane fouling propensity. Figure 6 shows a normalized water flux after a corresponding fraction of sodium alginate was added to FS. The water flux was evaluated under the PRO mode using 1 M NaCl DS. A corresponding amount of sodium alginate was added to the feed solution, and the lower water flux was normalized to initial values. To measure recovering propensity, sodium alginate DS was replaced with DI water after 300 min. After 300 min, the CZ5 membrane showed a higher percentage of water flux (47% in 250 mg/L sodium alginate, 42% in 1000 mg/L sodium alginate) than the CA membrane (37% in 250 mg/L sodium alginate, 29% in 1000 mg/L sodium alginate). When DS was changed to DI water for 90 min, the CZ5 membrane recovered 74 and 65% of its initial flux, while the CA membrane recovered 53% and 44% in 250 mg/L and 1000 mg/L sodium alginate, respectively. The modified surface properties may affect the propensity for alginate fouling by reducing the adhesion force. Also, the microstructure of the CZ5 membrane, represented by reduced structural parameters, may contribute to fouling resistance by hindering the sodium alginate accumulation in the porous support layer.

## Conclusion

In this work, we applied ZIF-302 nanoparticles to fabricate a CA base composite membrane to enhance osmotic water flux. The ZIF-302 showed water selectivity in brackish water by improved osmotic water flux. The 5 wt% of ZIF-302 loading enhanced pure water flux while maintaining water/ion selectivity, while higher loading spoiled water/ion selectivity. The enhanced osmotic water flux was attributed to higher pure water permeability as well as reduced structural parameters. Furthermore, the CA/ZIF-302 membrane showed enhanced alginate fouling resistance with higher water flux remaining in the PRO mode. The CA/ZIF-302 composite membrane was easily fabricated using the phase inversion method used in commercial membrane fabrication. These results show that ZIF-302 can be used to enhance performance and viability of CA membranes for ODMPs.

## Experimental

### Materials

CA (39.8 wt% acetyl, Mw ~30,000 g/mol) and sodium alginate (99%) were obtained from Sigma-Aldrich (USA). Sodium chloride (NaCl, 99.9% purity), magnesium chloride (MgCl_2,_ 99.9%) and n-methyl-2-pyrrolidone (NMP, 99.9%) were purchased from Alfa-Aesar. Acetone (99.9%) was purchased from Daejung Chemical Co. Ltd. (South Korea). All ZIF crystal fabrication was conducted as presented in previous work^36^. 5(6)-methylbenzimidazole (mbImH) and N,N-dimethylformamide (DMF) were purchased from the Sigma-Aldrich (USA), and anhydrous methanol was obtained from Daejeung Chemical Co. Ltd. (South Korea). 2-methylimidazole (2-mImH) and zinc nitrate tetrahydrate were purchased from Merck Chemical Co (Germany). All of these chemicals were used without further purification. All experiments were performed in air.

### Synthesis of ZIF nanoparticles and characterization

To synthesize ZIF-302, we used a mixed linker method, as described in previous work^36^. 2-mImH (9.9 mg, 0.120 mmol), mbImH (21.4 mg, 0.140 mmol), and Zn(NO_3_)_2_·4H_2_O (29.8 mg, 0.114 mmol) were added in a mixture of DMF (3.5 mL) and water (0.5 mL) in a 10-mL tightly capped vial. The solution was heated at 120 °C for 3 days. The colorless particles were precipitated and washed with 5 mL of DMF 3 times for one day.

After the DMF washing, as-synthesized ZIF samples were solvent exchanged 3 times per day with methanol at room temperature over 3 days. Collected samples were dried under vacuum at room temperature for 24 h, followed by heating at 180 °C for 2 h for activation.

As-synthesized ZIF-302 crystals were transferred into 20 ml glass vials with DI water, and sonicated for 10 min to immerse ZIF particles so they are under the glass vial. After bath sonication, the mixture was probe-sonicated for 4 h at 110 W power with a 3 s pulse and 1 s pause. Vials were placed on aluminum racks with a continuous supply of ice-water to prevent overheating. The ZIF solution was added to disposable plastic micro-cuvettes and characterized using a dynamic light scattering instrument (Zetasizer Nano-ZS90, Malvern Instruments). For membrane preparation, the ZIF particles were dried in an oven for 2 days at 80 °C to remove water and moisture. The crystal structure of the ZIF particles was characterized by powder X-ray diffraction (PXRD, D8 ADVANCE, Bruker Corporation, USA). Molecular bonding of the ZIF particles was characterized by FTIR spectra (IFS-66/S, Bruker Corporation, USA).

### Membrane fabrication and characterization

The ZIF-302/CA composite membranes were prepared by the phase inversion method. Following material contents in Table 1, CA/ZIF-302 mixture (18 wt%) was dissolved in NMP (77 wt%) and acetone (5 wt%) to achieve casting solution. ZIF-302 nanocrystals were added to the solvent and bath and sonicated for 30 min to achieve homogenous dispersion prior to dissolving the CA powder. The fully dissolved casting solution was left at an ambient condition for 24 h to remove air bubbles and prevent defect formation. The degassed casting solution was poured on a flat glass plate and applied by a casting knife with a 60 μm depth. The casting solution was partially evaporated for 30 s under atmospheric conditions and coagulated in tap water. The coagulated membranes were stored in tap water overnight to extract residual organic solvents from the membranes.

To characterize the morphology, each membrane was broken in liquid nitrogen to achieve clean fracture surfaces. The fractured membranes were freeze dried for 2 days to completely remove water all content. Cross surfaces were scanned by scanning electron microscopy (SEM, S-4000H, Hitachi, Japan) and top surfaces were scanned by field emission scanning electron microscopy (FESEM, JSM7500F, JEOL, Japan).

### Lab scale membrane performance evaluation

The effective area of the membranes was 12.56 cm^2^ and feed solution (FS) and draw solution (DS) flowed using commercial hydraulic pumps with a constant flow rate of 150 ml/min. The FO mode used an active layer facing feed solution (AL-FS) conditions for FS and DS flows on active layers and support layers, respectively. Pressure retarded osmosis (PRO) mode used an active layer facing draw solution (AL-DS) conditions for FS and DS flows on support layers and active layers, respectively. Each mode was chosen to investigate conditions to minimize the ICP that ions blocked in the support layer reduce the osmotic pressure gradient across the active layer.

The NaCl and MgCl_2_ solutions with different concentration (0.5, 1, 1.5, 2.0 M) were used as DS and deionized (DI) water was used as FS. Corresponding amounts of sodium alginate were dissolved in FS and bath sonicated for 12 h for the alginate fouling experiment. The pure water flux (*J*_*w*_) was determined by permeate volume per unit time and effective area.

$${J}_{w}=\frac{{\rm{\Delta }}{V}_{draw}}{{A}_{m}\cdot t}$$

where, Δ*V*_*draw*_ is the volume change of FS, *A*_*m*_ is the effective membrane surface area in the cross-flow area and *t* is time. The reverse ion flux (*J*_*s*_) was calculated using the initial and final FS ion concentrations. The FS ion concentration was determined using electrical conductivity measured by a conductivity meter (PCS Testr 35, Eutech, Japan).

$${J}_{s}=\frac{{c}_{1}{V}_{1}-{c}_{0}{V}_{0}}{{A}_{m}\cdot t}$$

Where, c_0_ and c_1_ are the initial and final FS concentrations, respectively, and V_0_ and V_1_ are initial and final volumes, respectively. The each evaluation was repeated 3 times for each membrane to obtain an average value. Each membrane sample was used 4 times in different draw solution concentration (0.5, 1, 1.5, 2.0 M) with single draw salt (NaCl or MgCl_2_) and mode (PRO or FO). In the fouling experiments, the membrane samples were used only once in a clean state and disposed.

The membrane performance parameters were characterized using the RO test. In RO test, the membranes loaded to commercial filter holder (HP 4750, Sterlitech, Kent, WA) filtered 10 mM NaCl aqueous solution applying 5 bar of hydraulic pressure. Following results of RO tests, water permeability (*A*), ion rejection ratio (*R*) and ion permeability (*B*) were calculated using following equations:

$$A=\frac{{\rm{\Delta }}V}{{A}_{m}\cdot {\rm{\Delta }}P\cdot {\rm{\Delta }}t}$$

$$R=(1-\frac{{C}_{p}}{{C}_{f}})$$

$$\frac{1-R}{R}=\frac{B}{A({\rm{\Delta }}P-{\rm{\Delta }}\pi )}$$

where *A*_*m*_ is the effective area of the membrane, Δ*P* is the applied hydraulic pressure, Δ*t* is time, Δ*π* is osmotic pressure across the membrane, and *C*_*p*_ and *C*_*f*_ are the ion concentrations of the permeated solution and feed solution, respectively.

Based on ICP theory introduced by previous work^51^, the structural parameter was calculated using following equation:

$$S=\frac{D}{{J}_{v}}\,\mathrm{ln}\,\frac{A\cdot {\pi }_{draw}-{J}_{v}+B}{A\cdot {\pi }_{feed}+B}\,$$

where *D* is the diffusion coefficient of the solute and *π*_*draw*_ and *π*_*feed*_ are the osmotic pressures of the draw solution and the feed solution, respectively.
